# Low-Temperature Degradation of Aflatoxins via Oxygen Plasma: Kinetics and Mechanism Driven by Atomic Oxygen Flux

**DOI:** 10.3390/ma18132924

**Published:** 2025-06-20

**Authors:** Nina Recek, Rok Zaplotnik, Gregor Primc, Peter Gselman, Miran Mozetič

**Affiliations:** 1 Jozef Stefan Institute, Jamova Cesta 39, 1000 Ljubljana, Slovenia; nina.recek@ijs.si (N.R.); rok.zaplotnik@ijs.si (R.Z.); gregor.primc@ijs.si (G.P.); 2 Interkorn Ltd., Gančani 34, 9241 Beltinci, Slovenia; peter.gselman@interkorn.si

**Keywords:** plasma, oxygen, surface, toxin, degradation, kinetics

## Abstract

Aflatoxins are toxic organic substances that are synthesized on the surfaces of seeds, nuts, and similar products by some fungi under elevated humidity. They decompose at temperatures well above 130 °C, so standard heating or autoclaving is an obsolete technique for the degradation of toxins on surfaces without significant modification of the treated material. Non-equilibrium plasma was used to degrade aflatoxins at low temperatures and determine the efficiency of O atoms. A commercial mixture of aflatoxins was deposited on smooth substrates, and the solvent was evaporated so that about a 3 nm thick film of dry toxins remained on the substrates. The samples were exposed to low-pressure oxygen plasma sustained by an inductively coupled radiofrequency (RF) discharge in either the E or H mode. The gas pressure was 20 Pa, the forward RF power was between 50 and 700 W, and the O-atom flux was between 1.2 × 10^23^ and 1.5 × 10^24^ m^−2^ s^−1^. Plasma treatment caused the rapid degradation of aflatoxins, whose concentration was deduced from the fluorescence signal at 455 nm upon excitation with a monochromatic source at 365 nm. The degradation was faster at higher discharge powers, but the degradation curves fitted well when plotted against the dose of O atoms. The experiments showed that the aflatoxin concentration dropped below the detection limit of the fluorescence probe after receiving the O-atom dose of just above 10^25^ m^−2^. This dose was achieved within 10 s of treatment in plasma in the H mode, and approximately a minute when plasma was in the E mode. The method provides a low-temperature solution for the efficient detoxification of agricultural products.

## 1. Introduction

Aflatoxins are toxic molecules produced by fungi, mostly the *Aspergillus* species [1,2,3]. Fungi produce aflatoxins under certain conditions, with sufficient humidity and warmth. Crop contamination can occur in fields, at harvest, or during storage. Storage under humid conditions is usually the main reason for the excessive contamination of food or feedstock with aflatoxins. If consumed, aflatoxins pose a significant risk to human and animal health, causing acute toxicity, weight loss, immune system suppression, tumor formation, liver cancer, etc. The detoxification of food products and removal of aflatoxins are essential for protecting human and animal health and preventing disease [1]. National standards limit the allowable concentration of aflatoxins in food and feedstock to roughly 2 and 20 µg per kg, respectively. If the contaminated materials are composed of spherical particles with a radius of 3 mm (like grains) and the toxins are evenly distributed on the surface, these concentrations correspond to toxin film thicknesses of roughly 0.003 and 0.03 nm in food and feedstock, respectively. Even a monolayer of toxins may be harmful.

Several methods have been proposed for the degradation of aflatoxins. The standard technique used for the degradation of organic materials is heating. However, aflatoxins have been reported to be very stable molecules. Therefore, the products contaminated with toxins are likely to be cooked before any degradation of aflatoxins occurs. Furthermore, prolonged heating is energy demanding and thus not useful for the treatment of large quantities of contaminated materials. Chemical degradation is always an option, but chemicals and/or residues may be as poisonous as toxins themselves. Thus, no solution that can be used for the routine decontamination of products contaminated with aflatoxins has been developed. Reviews of the techniques available for the degradation of aflatoxins were published several years ago [4,5,6,7,8,9,10,11,12,13]. Among them, plasma treatment is becoming increasingly popular [14,15,16,17,18,19,20,21,22,23,24,25,26,27,28,29,30]. All authors used atmospheric pressure plasmas for the degradation of aflatoxins. Such plasmas are useful for treating materials that cannot be evacuated, like vegetables [31,32] or liquids [33,34,35,36]. A pressure level below the saturated pressure of water vapor (approximately 30 mbar at room temperature) cannot be achieved because water will boil with any attempt to decrease the pressure below this natural limitation [15].

Grains, nuts, and similar materials release some water upon evacuation, but desorption is slow. Thus, pressures below a few Pa are usually easily achievable when treating reasonable quantities of such materials [19,37,38]. An advantage of low-pressure plasmas is their uniformity in a large volume and excellent energy efficiency because low-pressure plasma can be sustained at a power density as low as 1 W/L (1 kW/m^−3^) [39]. The reason for such excellent efficiency is the lack of gas-phase reactions. Therefore, practically all plasma species are available for interaction with solid materials [40,41]. Another advantage of low-pressure plasma is the availability of reliable probes for measuring the fluxes of various reactive species on the surfaces of treated materials [14,41,42].

In this article, we treated aflatoxins with low-pressure oxygen plasma. This presents the first systematic comparison of H/E-mode effects on aflatoxin degradation efficiency and establishes an oxygen-atom-dose–degradation kinetic model. The key objective was to determine the degradation versus the dose of O atoms and provide the efficiency, thus giving directions for upscaling and practical applicability.

## 2. Materials and Methods

### 2.1. Materials

A stock solution of the aflatoxin G2, G1, B2, and B1 mixture was purchased from Biopure (Romer Labs, Tulln an der Donau, Austria). The BiopureTM MIX contained 20 µg/mL aflatoxins in acetonitrile. A film of toxins was formed on a smooth glass surface by depositing a 2 µL droplet of the stock solution. The glass substrate was first cleaned in ethanol in an ultrasound bath and then activated by a brief treatment in oxygen plasma. A droplet of the stock solution was uniformly spread on a substrate with a diameter of 4 mm. The acetonitrile was left to evaporate from the surface under ambient conditions, leaving a fairly homogeneous film of toxins on the substrate surface. Taking into account the droplet volume (2 µL), the original toxin concentration (20 µg/mL), the size of the substrate (area of about 12 mm^2^), and the density of dried toxins of about 10^3^ kg/m^−3^, the thickness of the dry aflatoxin film was determined using a standard protocol [43] and was about 3 nm. All samples were made in triplicate.

### 2.2. Detection of Toxins

The aflatoxins used in our experiments exhibited fluorescence with a characteristic peak at 455 nm. The intensity of this peak was measured upon illumination with photons of higher energy. A multimode microplate reader (Tecan Infinite PRO 2000, Männedorf, Switzerland) was used. Fluorescence at an excitation wavelength of 365 nm and an emission wavelength of 455 nm was measured for untreated samples and samples treated under different conditions. We calibrated the fluorescence versus the thickness of the deposited toxin film. Different thicknesses were prepared by dilution of the original stock solution, and droplets of different aflatoxin concentrations in acetonitrile were deposited on glass substrates to measure the calibration curve.

### 2.3. Plasma Treatments

**Figure 1:**
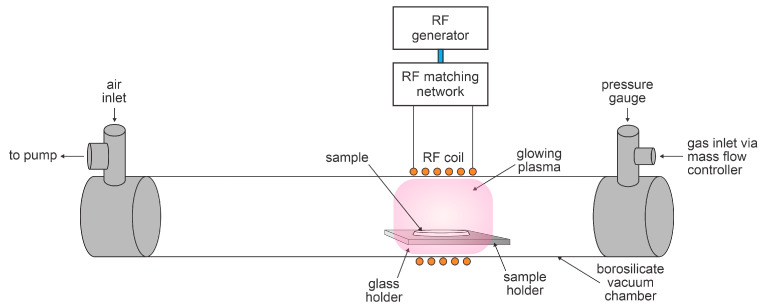
Schematic of the experimental setup for the degradation of toxins with oxygen plasma.

The samples with toxin coatings were treated with oxygen plasma in the discharge chamber of the system, which is illustrated in Figure 1. Plasma was inductively coupled with a radiofrequency (RF) generator via a water-cooled coil and a matching network optimized for coupling in the H mode. The discharge tube length was 800 mm and the inner diameter was 36 mm. The reactor was continuously pumped using a two-stage oil rotary vacuum pump with a nominal pumping speed of 80 m^3^/h. The pump was connected to the reactor at one end through a series of stainless-steel tees and a bellow, onto which a simple air inlet valve was mounted to vent the entire vacuum system. A gas inlet was connected to the discharge tube on the other side, which was equipped with a capacitive absolute pressure gauge (MKS Instruments, Andover, MA, USA). A six-turn water-cooled copper coil with a length of 70 mm was wrapped around the tube, as illustrated in Figure 1, and a Cesar 1310 RF power generator (Advanced Energy, Fort Collins, CO, USA) operating at 13.56 MHz was coupled to the coil via a matching network (Advanced Energy, Fort Collins, CO, USA). The generator operated at an adjustable output power of up to 1 kW. The generator is also equipped with a detector of reflected power, which can be large in the case of coupling in the E mode. A catalytic probe (Plasmadis, Ljubljana, Slovenia) was installed in the discharge tube to measure the density of neutral oxygen atoms.

## 3. Results and Discussion

### 3.1. Calibration of the Fluorescence Meter

**Figure 2:**
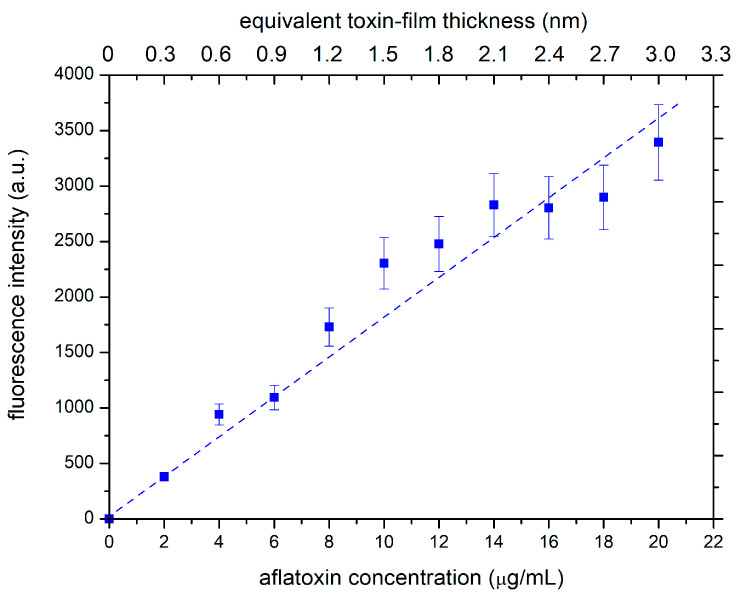
Fluorescence of the deposited and dried toxin films.

Calibration was performed by measuring the fluorescence signal of samples with different toxin film thicknesses. Different thicknesses were obtained by dilution of the stock solution before droplet deposition. After drying the deposited solution, samples with different thicknesses of the aflatoxin layer were illuminated with the same source of primary photons, and the fluorescence signal was measured. Figure 2 shows the measured fluorescence signal. The lower x-axis in Figure 2 represents the dilution of the stock solution used for depositing the droplet, and the upper x-axis is the calculated thickness of the dry toxin film in the approximation of a uniform distribution on the entire surface of the glass substrate. The orange line shows that the fluorescence increases linearly with aflatoxin concentration, which indicates that the film is optically thin. This result is not surprising, considering that the thickness of the dry toxin film is in the order of nanometers. Such a linear behavior is advantageous because it enables a rather precise determination of the evolution of the film thickness upon etching the toxins with oxygen plasma.

### 3.2. Thermal Degradation of Aflatoxins

**Figure 3:**
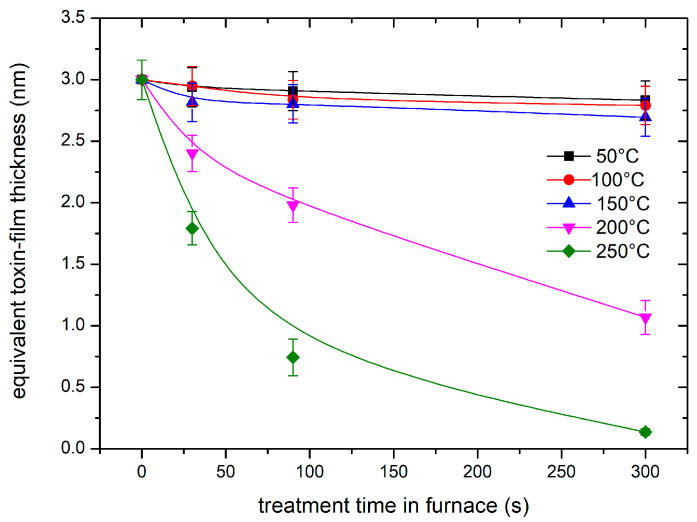
The thickness of the toxin film versus the treatment time in a furnace. The furnace temperature was used as a parameter.

Some samples with a dry film of aflatoxins with a thickness of 3 nm were placed in a massive furnace and heated at an initial rate of several 10 K/s. The furnace temperature was then adjusted to different values. A “steady temperature”—i.e., temperature not deviating more than ±3 K from the adjusted temperature—was achieved within 5–10 s. The samples were left at a selected temperature for the desired time, cooled to room temperature, and probed by fluorescence. Furnace temperatures of 50, 100, 150, 200, and 250 °C were selected, and the treatment durations at these temperatures were 30, 90, and 300 s. The thickness of the remaining toxin film is plotted in Figure 3. The error bars represent the standard deviation because all values were measured for three “identical” samples.

### 3.3. Calibration of the Plasma Source

Low-pressure inductively coupled RF oxygen plasma is an extensive source of neutral oxygen atoms. The dissociation fraction of oxygen molecules in such plasmas is many orders of magnitude larger than the ionization fraction, so the neutral O atoms are regarded as the main reactants upon treating organic matter with oxygen plasma [44,45]. Such oxygen plasma could be sustained in either the E or H mode, and the difference is explained in detail in [46]. Briefly, the E mode is capacitively coupled, so the impedance depends on the sheaths next to the powered and grounded electrodes, and the density of charged particles is rather low. The H mode uses conductive plasma as the secondary coil of the transformer, so the density of the charged particles should be large. There is an abrupt transition between the modes when changing either the gas pressure or the discharge power. The absorbed power at a given RF voltage is much larger in the H mode than in the E mode when using our matching network.

**Figure 4:**
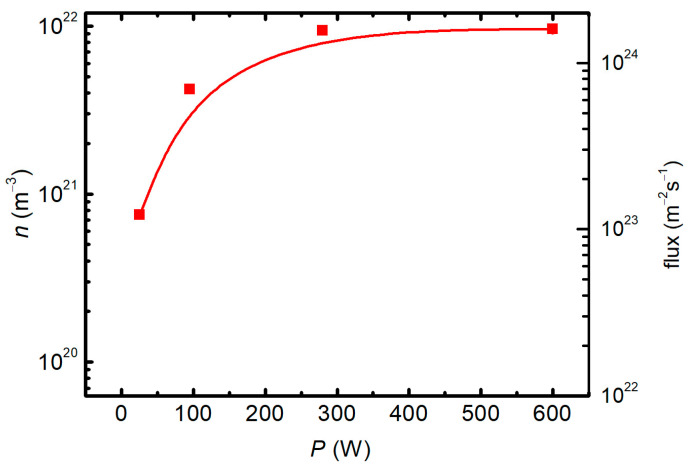
The O-atom density (left y-axis) and the flux (right y-axis) versus the real discharge power.

The density of O atoms was measured with a cobalt probe at different forward powers, and the reflected power was measured as well. The O-atom density versus the real power (the difference between the forward and reflected powers) is shown in Figure 4. The flux of O atoms was calculated using the standard formulae (i.e., *j* = ¼ *n* <*v*>) in the approximation of the room temperature and is also plotted in Figure 4.

### 3.4. Degradation Kinetics

Freshly prepared samples with the same amount of toxins, i.e., a toxin film thickness of 3 nm, were treated in the experimental system shown in Figure 1. A freshly prepared sample was mounted at the center of the RF coil, as shown in Figure 1. The vacuum system was evacuated to an ultimate pressure of 1 Pa, as measured by a vacuum gauge. Oxygen was introduced into the discharge tube and continuously pumped. The flow meter was adjusted to 100 sccm and an oxygen pressure of 20 Pa was established at the position of the vacuum gauge. Plasma was ignited by turning on the RF generator, which was pre-adjusted at a selected forward power. The discharge was turned on for a selected time. After turning off the RF generator, the oxygen flow was stopped and the system was slowly vented. The treated sample was probed by fluorescence within 10 min after the plasma treatment.

**Figure 5:**
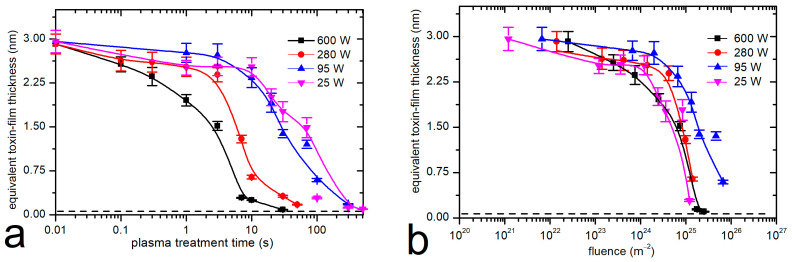
Thickness of the aflatoxin layers versus the plasma treatment time (**a**) and versus the dose of oxygen atoms (**b**); real discharge power is the parameter.

Systematic measurements were performed at four different discharge powers and eight different treatment times. The evolution of the remaining thickness of the toxin film, as probed by fluorescence using the calibration curve in Figure 2, versus the treatment time is shown in Figure 5a. The error bars indicate the standard deviation because all measurements were performed in triplicate. The discharge power was used as the parameter. The results shown in Figure 5a indicate a gradual decrease in the thickness of the aflatoxin layer with increasing plasma treatment duration. The thickness decreases more rapidly at a large power, which is explained by a larger flux of reactive oxygen species because the plasma at a large power is in the H mode. As expected, the degradation was slower when the plasma was in the E mode.

The fast degradation, as shown in the case of plasma in the H mode, could be explained either by degradation due to plasma species or simply because of the heating. As is shown in Figure 3, high temperatures cause degradation even in the absence of plasma. We could not measure the sample temperature in situ, but we estimated it as soon as we removed the sample from the discharge tube, i.e., within about 15 s after turning off the discharge. The sample, which was treated at a discharge power of 600 W, was heated to about 120 °C after treating it for 30 s. This is still a much lower temperature than that required for significant decomposition. For example, Figure 3 shows marginal degradation even after heating the toxin film at 150 °C for 300 s. Therefore, it can be concluded that the rapid degradation of the toxins upon treatment with oxygen plasma in the H mode is predominantly due to reactions with oxygen plasma species rather than the elevated temperature.

The degradation of toxins upon treatment with oxygen plasma in the E mode was slower. We also estimated the temperature of the sample after its removal from the plasma reactor, and it was only about 60 °C after treating the sample for 300 s at a power of 25 W. Obviously, the heating was marginal, and thermal effects could not be the reason for gradual degradation even in the E mode.

Figure 5b shows the same results as Figure 5a except that the thickness of the toxin film in Figure 5b is plotted versus the dose of O atoms. The measured points practically overlap in Figure 5b, especially when taking into account the accuracy of determination of the O-atom density, which was approximately 40%. The error arising from the application of the catalytic probe is due to several effects, including the effect of charged particles, the inaccuracy of the recombination coefficient, and the roughness of the catalytic material [47]. The overlap of the measured points in Figure 5b therefore indicates that the degradation is predominantly due to the interaction of aflatoxins with neutral oxygen atoms.

The exact mechanisms of the interaction between O atoms and aflatoxins have yet to be researched because of the complex structure [1]. Li et al. [48] provided a detailed analysis of the interaction between oxygen atoms and aflatoxins by molecular dynamics simulations. They studied the initial reactions to different parts of the AFB1 toxins. They found that oxygen atoms cause the opening of the furan ring by abstracting an H atom and, thus, the formation of an OH radical. An increased dose of O atoms may also cause the formation of an alcohol group. In the lactone ring, O atoms also undergo abstraction of an H atom, followed by reduction of the double C=C bond. In contrast, a double bond may be formed due to the abstraction of an H atom from the cyclopentenone. Formation of the CO_2_ molecule was found to be as a result of demethylation in the initial step of interaction between O atoms and the aflatoxin. This channel was found to be dominant in the fraction of the C-H bond breakage in a broad range of O-atom concentrations. The mechanisms proposed by Li et al. [48] are similar to those proposed for some organic materials with a simpler structure. For example, Ventzek’s team [49] provided a detailed model for interaction with polystyrene. They found that the initial reaction involved the substitution of an H atom in a polymer with an OH radical. As the surface becomes saturated with hydroxyl groups, further exposure to the O atoms causes the breaking of bonds in the aromatic ring. This is followed by the formation of carboxylic and ester groups, and finally, the formation of CO, CO_2_, and H_2_O molecules, and thus, etching by complete oxidation. Similar reactions probably occur on the surface of the aflatoxin film, but a theory on complete oxidation of the aflatoxins is yet to be developed.

The etching rate is rather low. As can be deduced from Figure 5b, the removal of the 3 nm thick film requires a dose of a few 10^25^ m^−2^. Taking into account these values, a rough estimation of the etching probability (i.e., probability that an O atom removes a carbon atom from the aflatoxin molecule) can be obtained:

(1) η=1723 (ρ x)/(mC DO),

where *ρ* is the density of dry aflatoxins, *x* is the initial thickness (before etching with plasma), *m*_C_ is the mass of a carbon atom, and *D*_O_ is the dose of oxygen atoms needed for the reduction in the fluorescence signal below the detection limit. The factor 17/23 takes into account the composition of the aflatoxin AFB1 (C_17_H_12_O_6_). Taking into account the numerical values (*ρ* = 10^3^ kg/m^3^, *x* = 3 nm, *m*_C_ = 1.7 × 10^−27^ kg, *D*_O_ = 3 × 10^25^ m^−2^), one can estimate the reaction probability *η* = 4 × 10^−5^.

The upper calculation is a very rough estimation that does not take into account the exact distribution and density of the dry layer of aflatoxins. The estimated probability represents a theoretical lower bound due to side reactions or surface adsorption losses. Furthermore, the value calculated using the approximation in Equation (1) is valid only if the layer of as-deposited toxins is uniformly thick and the etching is laterally homogeneous. Any analysis of the etching uniformity is beyond the scope of this article, but it is fair to mention it to avoid misinterpretation of Equation (1) and the corresponding value of the reaction probability.

In any case, the oxygen atoms enable effective degradation of the aflatoxin layer because the concentration remaining after achieving a dose of a few 10^25^ m^−2^ drops below the detection limit of the fluorescence method used in this study. According to Figure 2, the detection limit is close to the equivalent of about 0.03 nm in a uniformly thick film.

## 4. Conclusions

Oxygen plasma treatment causes degradation of aflatoxins at temperatures well below 100 °C. This is the temperature likely to be achieved when samples are treated in oxygen plasma sustained by an inductively coupled RF discharge in the H mode. A treatment time of about 10 s was sufficient to cause the remaining concentration of aflatoxins to decrease below the detection limit of the fluorescence analyzer. Prolonged treatment enables degradation, even at lower temperatures, provided that the plasma is sustained in the E mode. We measured the degradation curves for samples treated with weakly ionized oxygen plasma sustained at a discharge power as low as 25 W and observed degradation after treating the samples for a few minutes. The treatment time does not reveal much about the degradation, but we found that the dose of oxygen atoms was the decisive parameter governing the degradation. This was illustrated by the fact that all measured points were distributed on the same curve when plotting the residual toxins versus the dose of O atoms. The effective degradation of aflatoxins in oxygen plasma at low temperatures thus fulfilled the key objective of this study: to determine the degradation kinetics of aflatoxins as a function of the oxygen-atom dose.

We performed experiments with the as-received stock solution of the aflatoxin G2, G1, B2, and B1 mixture, so the thickness of the dried layer of toxins (after evaporation of the solvent) was about 3 nm. Such a film remains fairly intact at a low dose of oxygen atoms. Statistically significant degradation was observed after receiving a dose close to 10^24^ m^−2^. Degradation of the equivalent of a 3 nm thick film of aflatoxins was accomplished after receiving a dose of a few 10^25^ m^−2^.

Plasma sustained in the H mode achieves detoxification in 10 s. Taking into account the discharge power of 600 W and the surface covered with dense plasma in the H mode (roughly 100 cm^2^), the energy consumption is 60 J/cm^2^. This value could be lowered, perhaps by an order of magnitude, if larger systems were used as disclosed by the authors in [50]. Alternatively, one could use plasma in the E mode, provided the O-atom density is large, i.e., the discharge chamber is made from a material with a low coefficient for the heterogeneous surface recombination of O atoms. This fulfils the objective of evaluating efficiency for potential upscaling and confirms the scalability and energy-efficiency considerations that are critical for industrial applications. The comparison of E/H-mode effects on aflatoxin degradation efficiency indicates similar energy efficiency because the required treatment time is roughly an order of magnitude larger in the E mode, but the discharge power is an order of magnitude lower. In any case, the practical applicability is limited to large reactors capable of treating tons of grains per hour.

We estimated the reaction probability, i.e., the probability that an O atom causes the removal of a C atom from the sample surface, to about 4 × 10^−5^ using several approximations and simplifications, so this value should be taken just as a rough direction for any attempt to use oxygen plasma for the degradation of toxins on the surface of agricultural products. Namely, several approximations were taken into account, and the probe for measuring the flux of O atoms has a limited accuracy.

These results help in understanding plasma–toxin interaction mechanisms and provide a strong foundation for the development of practical, low-temperature, and energy-efficient plasma technologies for the detoxification of agricultural products contaminated with aflatoxins.

## Data Availability

The original data presented in the study are openly available in the repository Zenodo at https://doi.org/10.5281/zenodo.15586781.

## Abbreviation

The following abbreviation is used in this manuscript:

RF	Radiofrequency
